# Comprehensive analysis of small RNAs expressed in developing male strobili of *Cryptomeria japonica*

**DOI:** 10.1371/journal.pone.0193665

**Published:** 2018-03-12

**Authors:** Tokuko Ujino-Ihara, Saneyoshi Ueno, Kentaro Uchiyama, Norihiro Futamura

**Affiliations:** Department of Forest Molecular Genetics and Biotechnology, Forestry and Forest Products Research Institute, Forest Research and Management Organization, Tsukuba, Ibaraki, Japan; University of Naples Federico II, ITALY

## Abstract

Deep sequencing of small RNAs (sRNAs) in developing male strobili of second-generation offspring originating from a nuclear genic male sterile tree of *Cryptomeria japonica* were performed to characterize sRNA populations in the male strobili at early pollen developmental stages. Comparing to sequences of microRNA (miRNA) families of plant species and sRNAs expressed in the reproductive organs of representative vascular plants, 37 conserved miRNA families were detected, of which eight were ubiquitously expressed in the reproductive organs of land plant species. In contrast, miR1083 was common in male reproductive organs of gymnosperm species but absent in angiosperm species. In addition to conserved miRNAs, 199 novel miRNAs candidates were predicted. The expression patterns of the obtained sRNAs were further investigated to detect the differentially expressed (DE) sRNAs between genic male sterile and fertile individuals. A total of 969 DE sRNAs were obtained and only three known miRNA families were included among them. These results suggest that both conserved and species-specific sRNAs contribute to the development of male strobili in *C*. *japonica*.

## Introduction

*Cryptomeria japonica* (2n = 22) is a coniferous species endemic to Japan. Because of its excellent characteristics, *C*. *japonica* has been planted throughout the country; however, pollinosis induced by its pollen have become a major health issue in Japan [1]. The genes regulating the male strobili and pollen development in this species has attracted great interest; therefore, genes expressed in developing male strobili have been isolated and characterized [2]. In addition, several male sterile trees have been found [3–5], though their causative genes have not been isolated so far [6].

Small RNAs (sRNAs) regulate gene expression by degrading target mRNA, inhibiting translation, and modifying chromatin [7]. These regulatory sRNAs have been categorized into two major classes based on their origin, such as microRNA (miRNA) and small interfering RNA (siRNA). The involvement of miRNA or sRNA in the development of male reproductive organs and the regulation of male fertility has been reported in several angiosperm species [8–12]. Although the contribution of sRNAs to male fertility is unclear in coniferous species, the regulation pathway of reproductive organ development by several miRNAs were conserved in *Pinus tabuliformis* [13]. This suggested a role for sRNAs in the male reproductive organ development of coniferous species.

In this study, a comprehensive analysis of sRNAs expressed in the male strobili of *C*. *japonic*a was performed using high-throughput sequencing. The material trees were both male sterile and fertile trees derived from a naturally occurring male sterile tree ‘Toyama-1’, which carries a recessive male sterile gene “*ms1*” [14, 15]. We focused on sRNA expressed at the early pollen developmental stages because ‘Toyama-1’ expresses a defect in microspore development at the tetrad stage [16]. To our knowledge, this is the first report on the sRNA transcriptome of *C*. *japonica*; thus, the obtained sRNAs were first characterized based on sequence similarity to RNA sequences of plant species. The expression of these sRNAs were then compared between male sterile and fertile individuals. Our results provide clues to unravel the roles of sRNAs in the development of male reproductive organs of coniferous species.

## Materials and methods

### Plant materials

**Fig 1:**
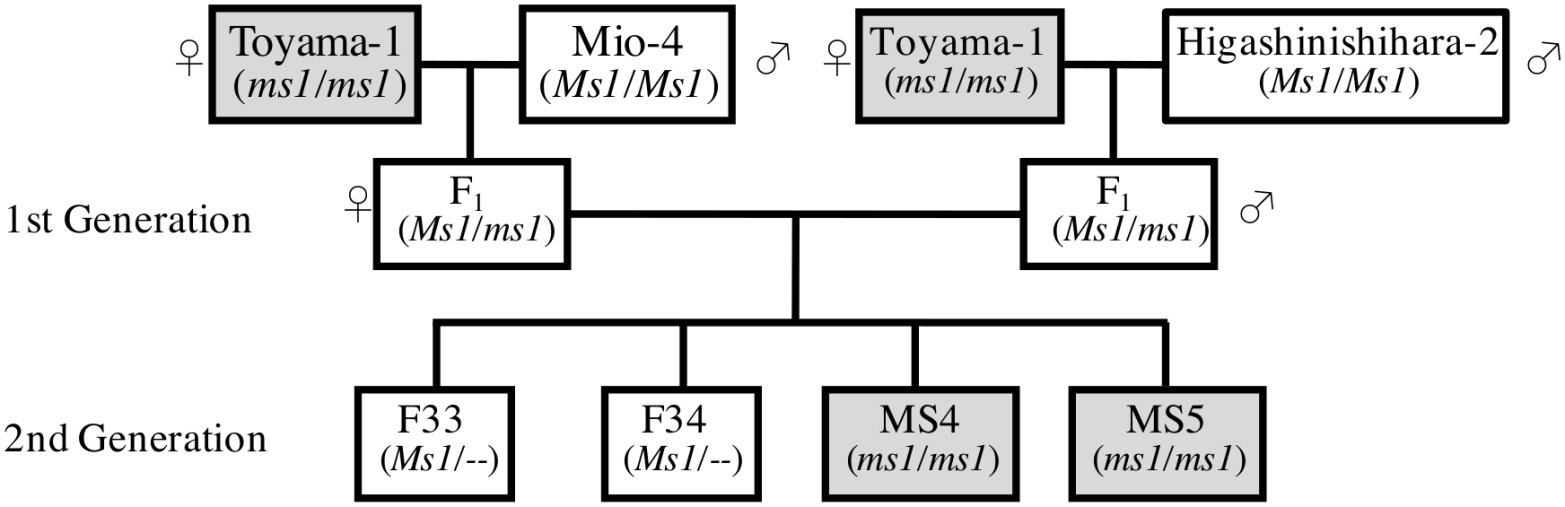
Pedigree chart for materials used in this study. Shaded squares represent male sterile individuals expected to be homozygous for *ms1*.

Two male sterile (MS04 and MS05) and two male fertile (F33 and F34) trees were used in this study. These trees were second-generation offspring originating from ‘Toyama-1’ (Fig 1). Under natural conditions, the microspore mother cells undergo meiosis from late September to mid-October, and pollen develops until December [5]. Male strobili were harvested on September 30th and October 4th, 7th, 19th in 2011, and on October 16th, 2012. The microspore developmental stage was assessed by examining 9–12 male strobili from each individual under a light microscope (S1 Fig). In 2011, male strobili were fixed in formalin–acetic acid–alcohol and stained with hematoxylin–eosin (HE). The developmental stage was not completely synchronized (S1 Table), even between microsporangia in the same male strobilus. In 2012, male strobili were crushed on a slide and microspores released from them were stained with a dye (ponceau 4R, KYORITSU FOODS CO.INC, Tokyo, Japan). Tetrads were observed in all individuals on October 16th, 2012.

### Sample collection and sRNA sequencing

Total RNA was isolated using plant RNA isolation reagent (Invitrogen, Carlsbad, CA, USA), according to a method modified to extract sRNAs [17]. In addition to sRNAs from male strobili, sRNAs isolated from needles of a fertile individual (F34) on May 17th, 2012 were used to construct an sRNA library. The RNAs were size-fractionated by acrylamide gel electrophoresis, and RNAs with 18–30 nucleotide (nt) in length were excised from the gel. In total, 20 sRNA libraries were generated from all but RNA from F34 on October 7th, 2011 and were subjected to sequencing analysis using HiSeq2000 (Illumina, San Diego, CA, USA). Library preparation and sequencing were conducted by Hokkaido System Science (Sapporo, Hokkaido, Japan).

### Sequence resources

The publicly available sequences used in annotating *C*. *japonica* sRNAs are listed in Table 1. Because the whole genome sequence is not yet available, the repetitive sequences of *C*. *japonica* were deduced from 1,185 contigs from 159 genomic BAC clones, by using RepeatScout [18] and LTR_finder [19] with default settings (S1 File). The BAC clones were derived from materials unrelated to ‘Toyama-1’ [20]. The predicted repeat sequences were compared with other plant repeat sequences using BLAST 2.2.26 (tblastx, e-value ≤1e-05). In order to detect pri-miRNA from transcripts of *C*. *japonica*, tentative contigs were generated from RNA-Seq data of male strobili at early pollen development stage (Table 1). These data were derived from individuals of T5 family, a backcross between ‘Toyama-1’ and a F1 hybrid of ‘Toyama-1’ and ‘Nakakubiki-4’ (male-fertile, *Ms1*/*Ms1*) [21]. Assembling was carried out using CLC Genomics Workbench 8 (Qiagen, Tokyo, Japan) for each sample. Generated contigs were further clustered by cd-hit-est with the “-c 0.98,” “-aS 1”, and "-aL 0.005" options [22]. Obtained 176,142 contigs were used for pri-miRNA detection.

**Table 1:** Sequence resources used in this study.

Description	Species	Reference	URL, Accession number
miRbase 21		[23]	http://www.mirbase.org/
Rfam 11.0		[24]	http://smallrna.danforthcenter.org/
TAIR10	*Arabidopsis thaliana*	[25]	ftp://ftp.arabidopsis.org/home/tair/Sequences/
Plant Repeat Database	Angiosperm species	[26]	http://plantrepeats.plantbiology.msu.edu/
sRNA sequences	land plants	[27]	http://smallrna.danforthcenter.org/, (S2 Table)
sRNA sequences	*Picea abies*	[28]	http://www.ebi.ac.uk/ena, ERP002476
Transposable elements	Coniferous species		https://www.ncbi.nlm.nih.gov/nucleotide/, (S3 Table)
Full-length cDNAs	*Cryptomeria japonica*	[2], [29]	https://www.ncbi.nlm.nih.gov/nucleotide/, AK406520-AK416748, AK416865, AK416866, and FX334350-FX347193
Transcriptome data of male strobili	*Cryptomeria japonica*		http://trace.ddbj.nig.ac.jp/dra/index.html, DRA006304
Complete genome sequence of chloroplast	*Cryptomeria japonica*	[30]	https://www.ncbi.nlm.nih.gov/gene/, NC_010548.1
Repeat Sequences	*Cryptomeria japonica*		(S1 File)

### Analysis and annotation of sequencing data

Raw reads were processed to remove adaptor and low-quality sequences. Unique sequences of 18–30 nt in length were collected and counted using CLC Genomics Workbench ver. 5.5 (Qiagen). Pairs of sequences were not assembled in this process even a shorter sequence was perfectly aligned to a longer one, because such miRNAs can be originated from independent loci [31]. Low-complexity sequences were further filtered using the “Filter” tool in UEA sRNA Workbench v2.4.2 [32] and an in-house perl script in which sequences were filtered when a single nt accounted for ≥75% of the nucleotide composition. All unique sequences were deposited at the DDBJ Sequence Read Archive (DRA) under the accession number DRA005886. Normalized count data of each sRNA were calculated as counts per million (cpm) values using the cpm function in edgeR [33]. sRNAs with the cpm value 10 or higher in at least one sample were retained for further analysis as “retained_sRNAs” (S2 Fig).

Retained_sRNAs were compared with sequences from other species using oligomap to save computation time [34]. Additionally, retained_sRNAs were compared with plant miRNAs in miRbase21using BLAST 2.2.26+ (blastn-short, the word size of 7, e-value ≤0.001). Putative miRNA precursors (pri-miRNAs) of *C*. *japonica* were screened using mireap (http://sourceforge.net/projects/mireap/). Secondary structure of predicted miRNAs was carried out by mfold program [35]. Target genes of retained_sRNAs were predicted using the psRNAtarget [36], with the length for complementarity scoring of 18 and the maximum expectation values of 2.0. Putative functions of predicted target genes were deduced by blastx against TAIR10 proteins with a significance threshold of 1e-05 [25].

### Comparing the expression patterns of sRNAs between samples

In the hierarchical clustering of samples based on the log-transformed cpm value of each sRNAs, the distance between the samples was calculated using the Dist function in the R package “amap”, with the spearman method. Samples were then clustered using the R hclust function.

Differentially expressed (DE) sRNAs between male sterile and fertile samples on the same sampling date were detected using pairwise exact test in edgeR [33]. sRNAs with false discovery rate (FDR) ≤0.01 were considered to be significant. Significant sRNAs were accepted as DE sRNA candidates if both the samples in the upregulated group (male sterile or fertile) have a cpm value 10 or higher. A heatmap was generated using log-transformed cpm values using the heatmap.2 function in the R package “gplot.” The distance between each DE sRNA was calculated in the same way as done for the distance between each sample as described above.

### Quantitative real-time polymerase chain reaction analysis

The expression levels of the DE sRNAs were examined by quantitative real-time reverse transcription polymerase chain reaction (qRT-PCR) using the High-Specificity miRNA QRT-PCR Detection Kit (Agilent Technologies, Santa Clara, CA, USA) according to manufacturer’s instruction. 5.8S rRNA was used as a reference gene for internal normalization. For putative target genes, qRT-PCR was carried out using KOD SYBR qPCR/RT Set III (Toyobo Life science, Osaka, Japan). Expression levels of putative target genes were normalized to the expression of Elongation factor 1 alpha (EF-1a). In both analysis, total RNAs of samples harvested on October 4th, 2011, (approximately 300ng each) were converted to cDNAs and were used in qRT-PCR as templates. Specific primer pairs of putative target genes and EF-1a of *C*. *japonica* were designed using primer3 [37]. Primer sequences used in qRT-PCR was listed in S4 Table. All PCR reactions were performed on a Light Cycler 480 (Roche, Mannheim, Germany) with three technical replicates. The crossing points were determined by 2nd derivative maximum method. Relative expression levels of each sRNA or target gene were calculated from the average of three technical replicates using 2^-ΔΔCT^ method [38].

## Results and discussion

### Collecting *C*. *japonica* sRNAs expressed in male strobili

A total of 325,801,966 reads obtained from 20 samples were filtered, combined and made non-redundant, resulting in 18,094,812 unique sequences. Among unique sequences, 14,094,827 sRNAs (77.9%) were singletons across the 20 samples analyzed. To focus on the sRNAs with a relatively high expression level, a total of 27,275 sequences with the cpm value 10 or higher in at least one sample were retained.

**Fig 2:**
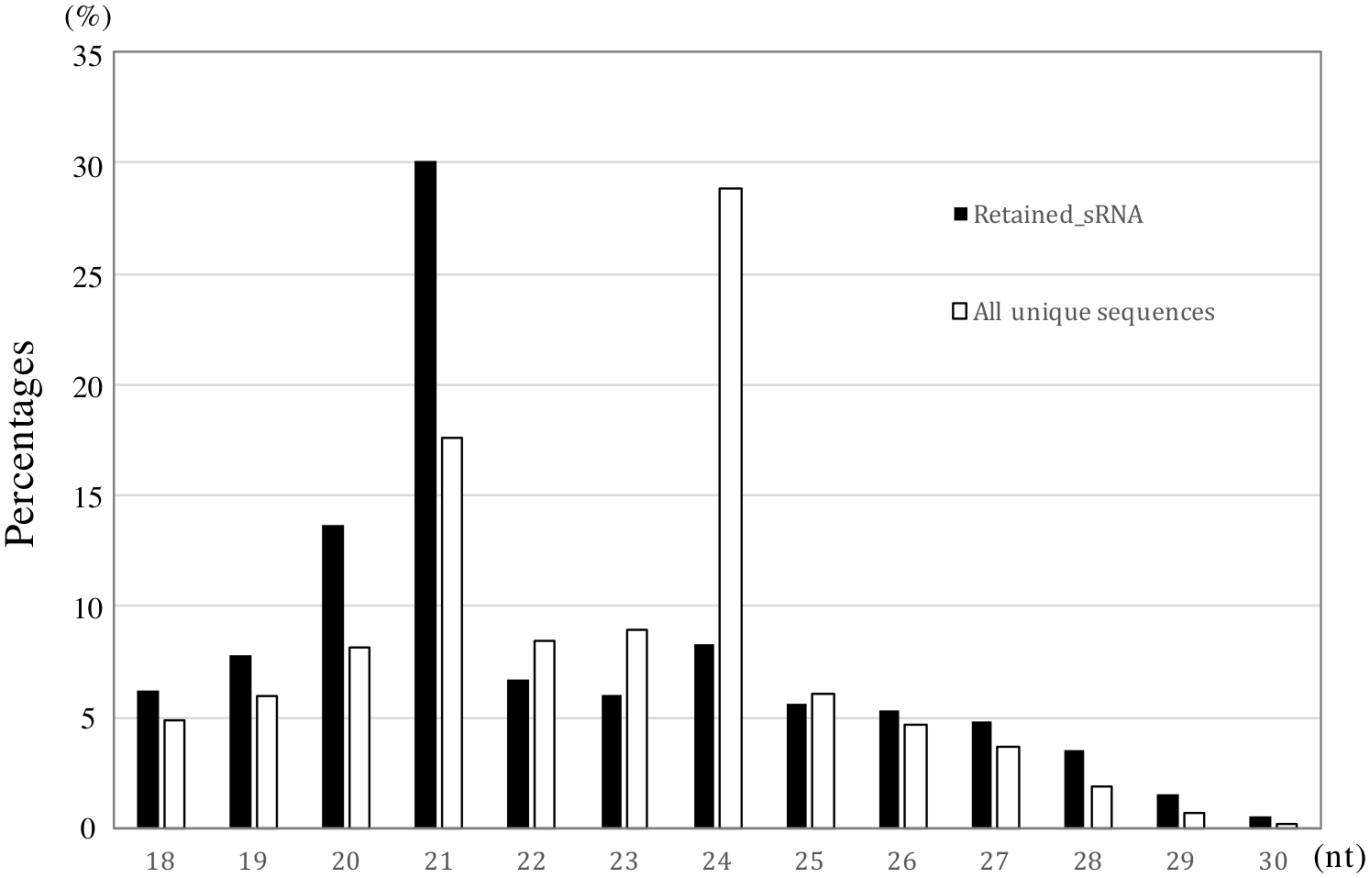
Length distribution of *Cryptomeria japonica* small RNAs (sRNAs). Frequency of each size class is shown as a percentage of the total number of retained_sRNAs (black bars) and all unique sequences (white bars).

The length distributions of all unique sequences and retained_sRNAs are shown in Fig 2. The most frequent size class of sRNAs was 24 nt, followed by 21 nt among all unique sequences. However, the most abundant class was 21 nt among the retained_sRNAs. This indicated that the 24 nt class included highly divergent sequences. The tissue specific expression of 24 nt sRNAs in male reproductive organs was reported in the *Picea abies* [28], but the 24 nt sRNAs are not much abundant in male strobili compared with needle samples in *C*. *japonica*. High expression of 24 nt sRNAs in the male reproductive organs might be characteristic to species in the Pinaceae family.

### Detecting phylogenetically conserved miRNAs expressed in the reproductive organs

By comparing retained_sRNAs to known plant miRNAs, 37 miRNA families consisting of 272 sRNAs were detected in *C*. *japonica* (Table 2). The retained_sRNAs were further compared to sRNAs expressed in the reproductive organs of a wide range of plant species [27] (S2 Table). Allowing one mismatch, 2,192 out of 27,275 sRNAs (8.0%) were ubiquitously expressed in the reproductive organs across the 27 compared species compared. Among them, 115 sRNAs belong to eight conserved miRNA families (miR156, miR159, miR166, miR167, miR168, miR390, miR396, miR894). It supported their ancient origin as suggested in Montes et al. [27]. These miRNA families likely have conserved roles in the development of reproductive organs of land plant species.

**Table 2:** Conserved microRNA (miRNA) families detected in *Cryptomeria japonica*.

miRNA family	No. of sRNA	Abundance^a^
miR166	83	1753786.94
miR396	15	56128.30
miR2916	49	26644.90
miR894	14	17689.60
miR319	5	14927.70
miR6478	16	6648.76
miR156	9	6404.87
miR1083	6	5559.05
miR162	4	5317.09
miR393	4	4104.35
miR159	8	2293.10
miR167	2	1582.96
miR482	9	1494.14
miR6300	6	1184.13
miR535	1	1135.35
miR5636	4	805.26
miR8155	1	801.55
miR169	3	727.36
miR858	1	714.57
miR390	1	671.37
miR172	3	669.00
miR168	2	635.59
miR398	3	616.36
miR5139	5	469.06
miR171	2	364.73
miR6725	4	245.04
miR1314	1	232.91
miR1030	1	219.44
miR2118	1	212.17
miR399	2	181.42
miR5298	1	122.89
miR5771	1	87.13
miR408	1	70.70
miR8562	1	41.99
miR845	1	41.42
miR6024	1	34.36
miR9781	1	18.17

^a.^ Abundance is shown as the total cpm of small RNAs (sRNAs) included in the same miRNA family.

In addition, a total of 170 sRNAs were common in the male reproductive organs of the compared gymnosperm species, whereas they were absent in angiosperm species. Majority of these sRNAs seemed to be novel, but three sRNAs were similar to miR1083, which was reported to be specifically abundant in gymnosperm species [27]. The sequence conservation and the relatively high expression may suggest a common role for miR1083 in male reproductive organ of gymnosperm species, although the expression of miR1083 is also reported in other organs [39].

### Identification of miRNA in transcripts of *C*. *japonica*

Previous study showed that the majority of miRNA sequences were found in only one or few species [40]. Therefore, we attempted to detect novel miRNAs of *C*. *japonica*. For this purpose, putative miRNA precursors among 23,057 *C*. *japonica* full length cDNAs (FLcDNAs) and 176,142 tentative contigs derived from male strobili were surveyed using mireap and it resulted in the prediction of 212 miRNA candidates. Many of these miRNA candidates were 21nt in length (68.4%, S3 Fig). Thirteen putative miRNA showed significant similarity to nine known miRNA: miR166, miR319, miR396, miR482, miR1083, miR1314, miR2118, miR5771, and miR6725. Hairpin structure, which is one of characteristics of pri-miRNA, was predicted from each corresponding sequence (S4 Fig). The remaining 199 predicted miRNAs (94.8%) did not show significant similarity to known miRNA sequences. While most of these novel miRNAs seemed to be specific to *C*. *japonica*, three of them expressed in reproductive organ of more than 20 plant species compared in this study. Interestingly, these three phylogenetically conserved miRNA sequences are present in organelle genome of plant species (S5 Table). Recent studies showed that part of sRNAs derived from chloroplast DNA (cpDNA) are footprints of pentatricopeptide repeat (PPR) proteins that play a role in organelle RNA processing. Two of three putative miRNA found above is derived from chloroplast and one of them may be the PPR binding site in terms of their location in chloroplast genome [41]. On the other hand, another putative miRNA located in intergenic region of cpDNA and corresponding pri-miRNA included hairpin form. Because the whole genome sequence of *C*. *japonica* is not yet available, we cannot clarify the origin of the putative miRNA here. The predicted miRNA will be one of the important target in future analysis.

Among the predicted novel miRNAs, six of the most ten abundant miRNA had weak similarity to miR482. The miR482 family is known to trigger the phased generation of siRNAs (phasiRNA) from NBS-LRR genes [42]. The function of miR482 family in reproductive organ development is unclear, but the miR482 family triggers phasiRNA production from noncoding transcripts in the reproductive tissues of monocots [43] and male cone of *P*. *abies* [44]. High abundance of miR482 family in male strobili of *C*. *japonica* suggested the specific phasiRNA production from noncoding transcripts in male reproductive organs might be common among coniferous species.

Albeit 37 putative mature miRNAs were found in the sRNA data (Table 2), pri-miRNAs for 28 miRNAs were not predicted. One explanation for this result is that FLcDNAs and tentative contigs were not derived from materials used in this study. Alternatively, some miRNAs might have rapid turnover and the consequently low pri-miRNA abundance among all transcripts.

### sRNAs mapped to repeat sequences of *C*. *japonica*

Parts of sRNAs are derived from repetitive sequences in the genome and act in an siRNA-directed silencing of transposable elements (TEs). Not only siRNAs, but recent studies showed that the miRNA are also involved in TE silencing [45]. Silencing the TEs during the reproductive organ development is important for maintaining genome integrity [46]; therefore, we screened sRNAs mapped to repeat elements of *C*. *japonica*.

A total of 2,306 repetitive sequences were predicted using BAC contigs of *C*. *japonica* with the mean length of 916 bp (S1 File). Allowing one mismatches, 171 (0.6%) retained_sRNAs were mapped to predicted repetitive sequences. The most abundant size class of mapped sRNAs was 24 nt (S5 Fig) and it corresponds to the fact that the sRNAs derived from the TEs are typically 24 nt [47]. Among the sRNAs mapped to predicted repeat sequences, putative miR5139, miR8155, and miR894 were found (Table 3). miR5139 and miR8155 belong to miR1511 family and contain the primer binding site (PBS) for tRNAi^Met^. The PBS is involved in the initiation of reverse transcription of LTR retrotransposons and believed to work in silencing retrotransposons [48]. Putative miR5139 and miR8155 had conserved nucleotides across miRNA harboring a PBS complement (Table 3). These miRNAs might participate in miRNA-mediated silencing of retrotransposons in *C*. *japonica*. miR894s showed abundant expression, but the function of miR894 in regulating TEs has not yet been reported as far as we know. miR894 was first reported in moss [49] and is expressed in reproductive organ of a wide range of plant species. It cannot be concluded here whether miR894 is involved in silencing retrotransposons but it is an intriguing question.

**Table 3:** The small RNAs (sRNAs) related to the predicted repeat sequences of *Cryptomeria japonica*.

Sequence^a^	Abundance^b^	Length (bp)	Annotation of mapped repeat sequences	miR family
GTTTCACGTCGGGTTCACCA	530.20	20	retrotransposon (gypsy)	miR894
TTTCACGTCGGGTTCACC	269.01	18	retrotransposon (gypsy)	miR894
TTTCACGTCGGGTTCACCA	2915.28	19	retrotransposon (gypsy)	miR894
TTCACGTCGGGTTCACCA	13000.91	18	retrotransposon (gypsy)	miR894
AAACCTG**GCTCTGATACC**	32.99	18	retrotransposon (copia)	miR5139
AACCTG**GCTCTGATACCA**	854.23	18	retrotransposon (copia)	miR8155
ATTCACGTCAGGTTCACC	122.91	18	retrotransposon (gypsy)	miR894
GTTTCACGTCAGGTTCACCA	79.00	20	retrotransposon (gypsy)	miR894
TTTCACGTCAGGTTCACCA	1520.97	19	retrotransposon (gypsy)	miR894
TTCACGTCAGGTTCACCA	3794.54	18	retrotransposon (gypsy)	miR894
TTTCACGTCAGGTTCACC	283.21	18	retrotransposon (gypsy)	miR894
GATTCACGTCGGGTTCACCA	124.56	20	retrotransposon (gypsy)	miR894
ATTCACGTCGGGTTCACCA	935.07	19	retrotransposon (gypsy)	miR894
ATTCACGTCGGGTTCACC	114.86	18	retrotransposon (gypsy)	miR894
TTCACGTTGGGTTCACCA	45.01	18	retrotransposon (gypsy)	miR894

^a.^ PBS complement sequence is indicated in bold.

^b.^ Abundance is shown as the total cpm of all samples analyzed.

### DE sRNAs between male sterile and fertile individuals

**Fig 3:**
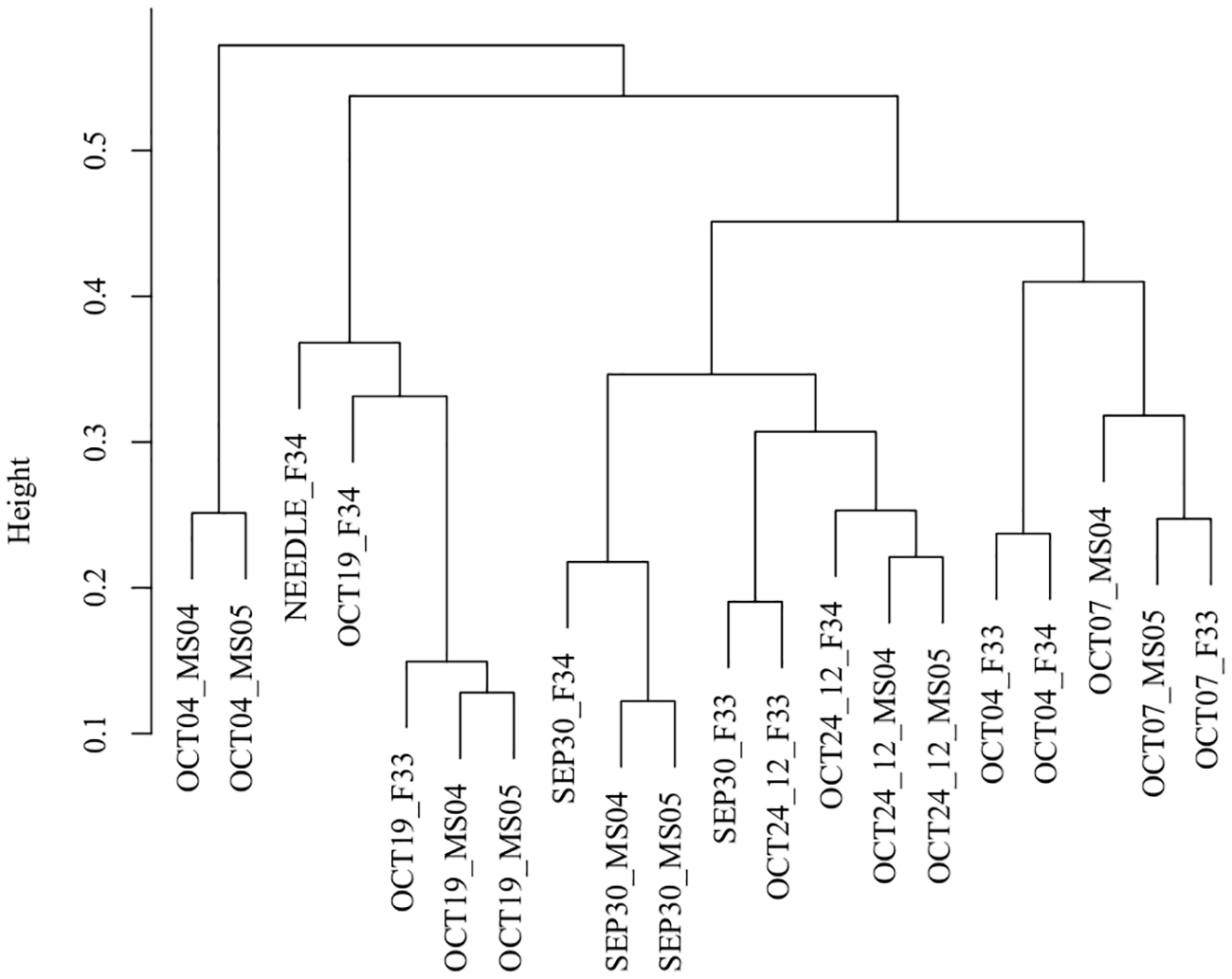
Clustering based on expression patterns of the retained_sRNAs. Prefix indicates sampling date.

**Fig 4:**
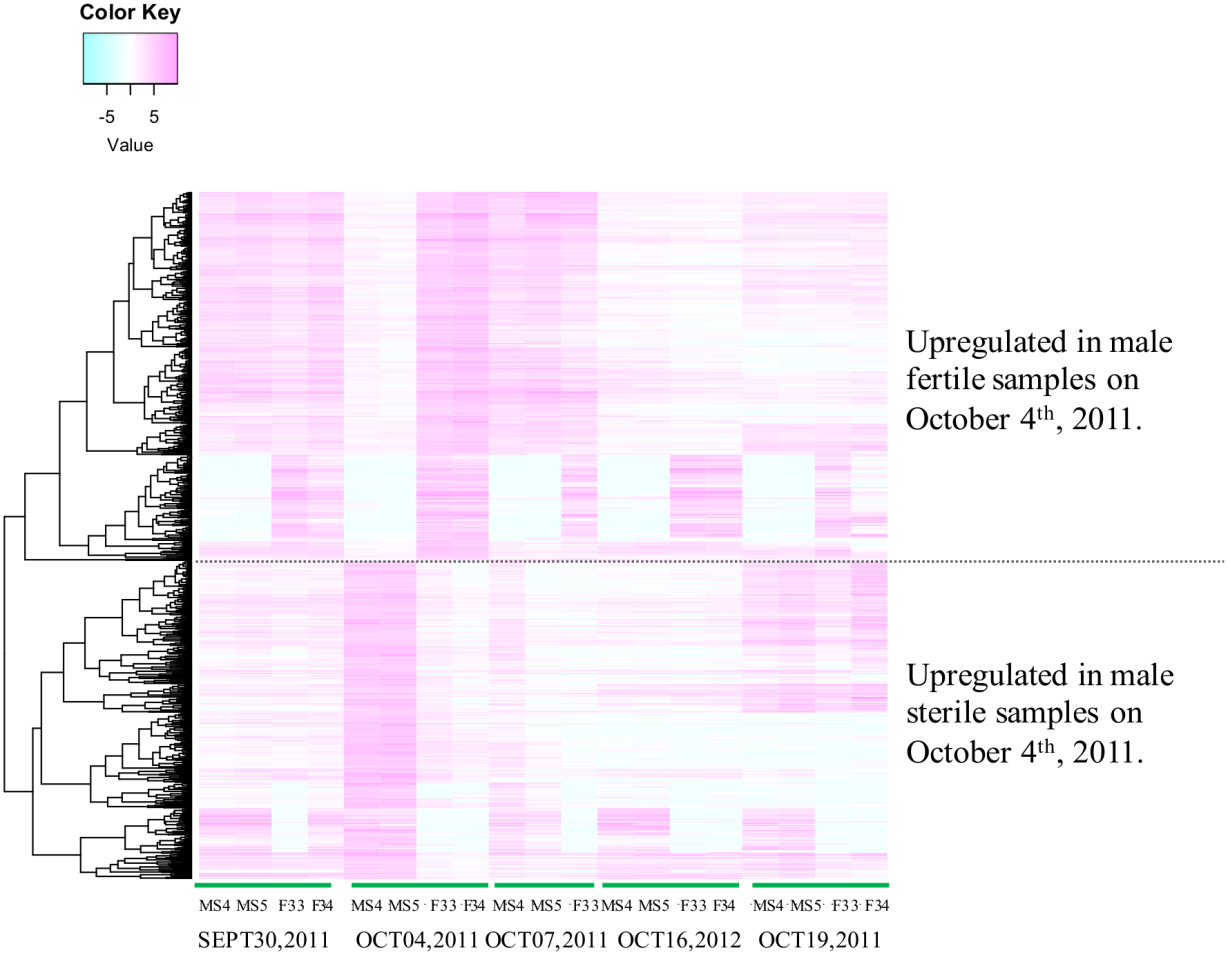
Expression profiles of DE sRNAs. Rows show sRNAs, and columns show samples. The color indicates the expression level of sRNAs (magenta indicates high, white indicates intermediate, and blue indicates low).

The clustering analysis showed that male sterile samples harvested on October 4th, 2011, had a distinct expression profile of sRNAs from other samples (Fig 3). By comparing sRNA expression between samples harvested on the same date, 969 DE sRNA candidates were detected (Table 4, Fig 4). The high number of specific DE sRNAs in samples on October 4th, 2011, may reflect the large change in gene regulation caused by male fertility and/or by the developmental stage. It should be noted that DE sRNAs detected here could arise from not only the differences in fertility but also the developmental stage because microspore development was not synchronized (S1 Table).

**Table 4:** The numbers of DE sRNAs at each sampling date.

Sampling date (MM/DD/YYYY)	Number of sRNA	
	Total	Specific^a^
09/30/2011	18	0
10/04/2011	906	807
10/07/2011	41	0
10/19/2011	23	10
10/16/2012	102	26

^**a**.^ DE sRNAs specific to the corresponding sampling date.

Among DE sRNAs, three, two, and one sRNAs showed sequence similarity to known miRNA, miR894, miR166, and miR5771, respectively. DE miRNAs, except for miR5771, are also expressed in the reproductive organs of most plant species [27]. Considering two of them mapped to predicted TEs, miR894 might be rather involved in the regulation of TEs than in the regulation of male strobili development. Upregulation of miR166 family has been reported in microspores of male sterile tomato [50]. To our knowledge, the role of miR5771 in the reproductive organs has not yet been reported. Several miRNA families obtained in this study, such as miR156, miR159, miR167, miR172, and miR319, were related to establishing fertility in angiosperm species [10,51–54]. However, their expression patterns were not significantly different by the test conducted in this study. Among putative novel miRNA predicted in this study, sixteen miRNAs specific to *C*. *japonica* were differentially expressed.

The male sterility of *ms1* is caused by a defect in the early stages of pollen wall development as described earlier. The core components of the pollen wall development pathway are likely conserved in *C*. *japonica* [5]. Target genes could be predicted for 547 DE sRNAs, and three sRNAs target homologous genes involved in early stages of pollen wall development of *Arabidopsis* (S6 Table), SHT [55] and IRX9-L/*SPONGY2* [56]. Two sRNAs were not known miRNAs, though one of which targets IRX9-L homolog was putative miRNA. qRT-PCR was carried out to verify the expression of these DE sRNA candidates. One sRNA (DEsRNA3) which targets SHT homolog showed higher expression in male sterile individuals as the result based on high-throughput sequencing (S6 Fig). However, the expression patterns for the remaining two DE sRNA based on the qRT-PCR results disagreed with the result of high-throughput sequencing. The results obtained here were rather preliminary because of small sample number, thus the reason of disagreement should be further assessed in future analysis. Transcripts expression levels of IRX9-L and SHT homolog are not negatively correlated to the corresponding DE sRNA expression levels inferred from qRT-PCR or high-throughput sequencing (S6 Fig). These genes might be regulated by translational inhibition rather than transcriptional cleavage, if it is actually regulated by the DE sRNA.

Although further analysis with more samples is needed to confirm target genes of obtained DE sRNAs and to elucidate whether DE sRNAs are involved in the regulation of pollen wall formation, the results obtained here provide clues for dissecting the function of sRNAs in the development of male reproductive organs in *C*. *japonica*.

## Supporting information

S1 Fig Light micrographs of pollen development. a. Early microsporocyte stage, b. Pre-meiotic stage, c. Meiotic stage, d. Tetrad stage, e. Microspore stage. f. Unseparated tetrads in a male sterile individual (MS04) at microspore stage.
(PPTX)

S2 Fig Flowchart of the sRNA data processing and the miRNA candidates detection. (PPTX)

S3 Fig Length distribution of predicted miRNA of *C*. *japonica*. (PPTX)

S4 Fig Secondary structure of predicted pre-miRNA hairpins of *C*. *japonica*. The dominant mature miRNAs were indicated by green letters.
(PPTX)

S5 Fig Length distribution of retained_sRNAs mapped to predicted repetitive elements of *C*. *japonica*. (PPTX)

S6 Fig Quantitative PCR of DE sRNA candidates and their putative targets in *C*. *japonica*. Normalized expression level in MS04 sample were arbitrarily set to 1. “qPCR” indicates sRNA expression level based on qRT-PCR and “CPM” indicates the relative expression level based on normalized count data obtained by high-throughput sequencing. For the qRT-PCR result, values are the mean of three technical replicates. Predicted target gene was cjIRX-9 for DEsRNA1 and DEsRNA2, and cjSHT for DEsRNA3.
(PPTX)

S1 File Predicted repeat sequences of *C*. *japonica*. (FAS)

S1 Table Major developmental stage observed in each individual. a. Also see S1 Fig for details about the developmental stage. b. Sample was not subjected to sequencing analysis.
(XLSX)

S2 Table Origins of reproductive organ sRNA data. a. Montes et al. 2014. b. Nystedt et al. 2013.
(XLSX)

S3 Table Repeat element sequences of coniferous species used in this study. (XLSX)

S4 Table Primer sequences used in qRT-PCR. (XLSX)

S5 Table Novel and phylogenetically conserved miRNA candidates. (XLSX)

S6 Table DE sRNAs and their target genes involved in pollen development. a. Locus name for *Arabidopsi*s genes homologous to predicted target genes of *C*. *japonica*.
(XLSX)
