# Supplementary material for: Comprehensive analysis of small RNAs expressed in developing male strobili of Cryptomeria japonica
Source: PLoS One. 2018 Mar 12;13(3):e0193665. doi: 10.1371/journal.pone.0193665 (PMC5846777; doi:10.1371/journal.pone.0193665)
Supplement: S1 Fig — a. Early microsporocyte stage, b. Pre-meiotic stage, c. Meiotic stage, d. Tetrad stage, e. Microspore stage. f. Unseparated tetrads in a male sterile individual (MS04) at microspore stage. (PPTX) [file pone.0193665.s001.pptx]

## Slide 1
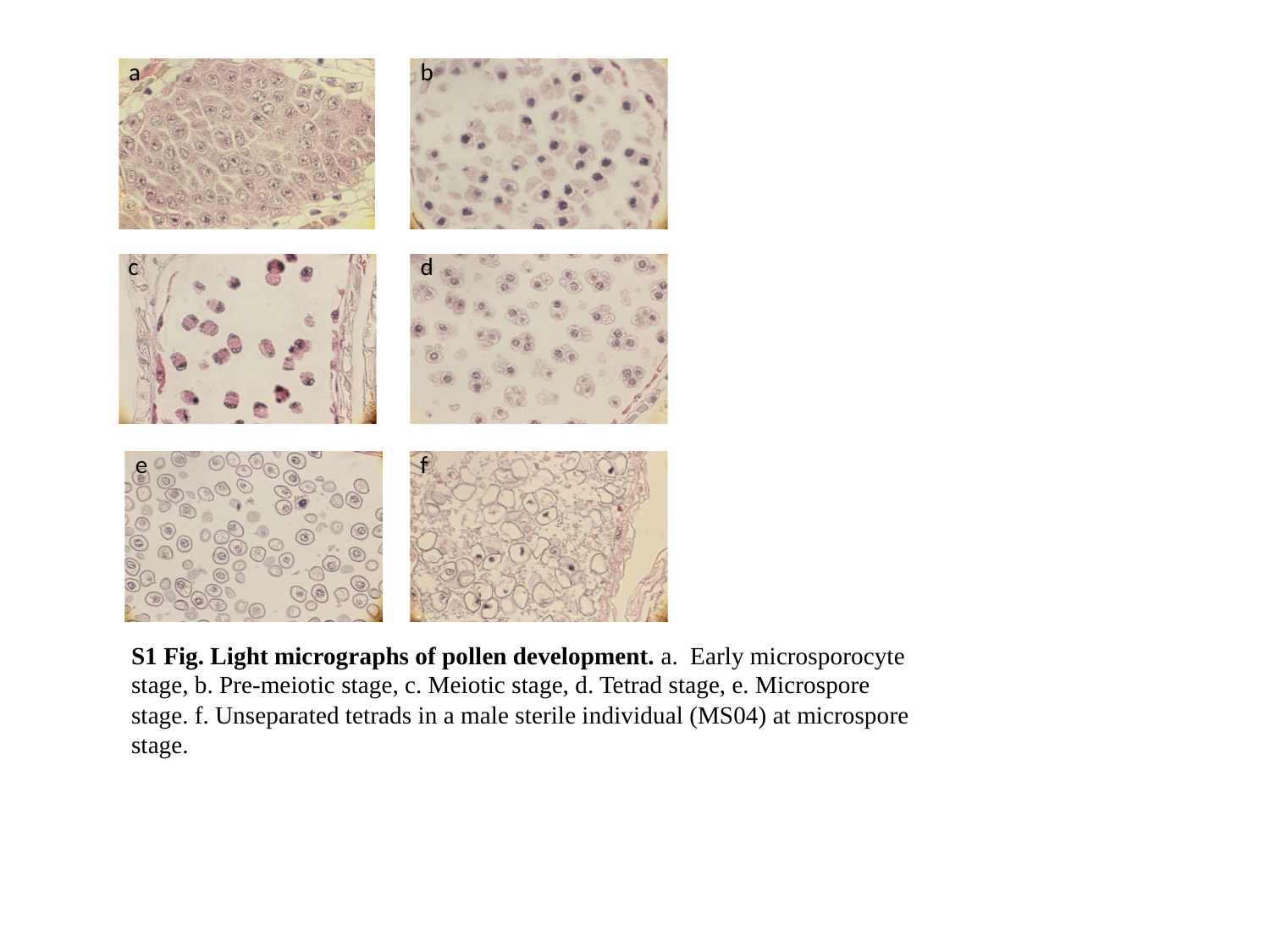

a
b
c
d
e
f
S1 Fig. Light micrographs of pollen development. a. Early microsporocyte stage, b. Pre-meiotic stage, c. Meiotic stage, d. Tetrad stage, e. Microspore stage. f. Unseparated tetrads in a male sterile individual (MS04) at microspore stage.
