# Supplementary material for: Comprehensive analysis of small RNAs expressed in developing male strobili of Cryptomeria japonica
Source: PLoS One. 2018 Mar 12;13(3):e0193665. doi: 10.1371/journal.pone.0193665 (PMC5846777; doi:10.1371/journal.pone.0193665)
Supplement: S2 Fig — (PPTX) [file pone.0193665.s002.pptx]

## Slide 1
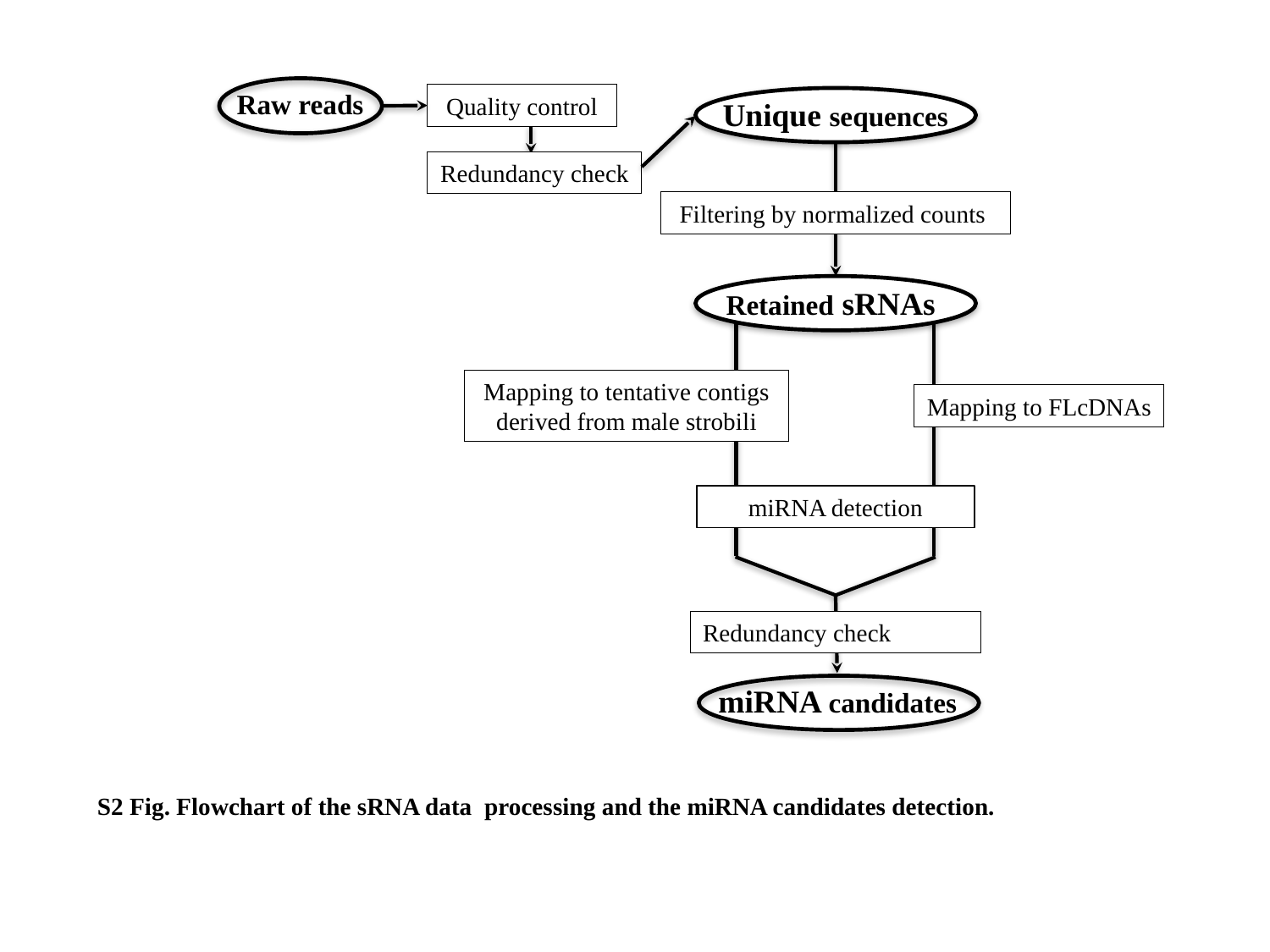

Raw reads
Quality control
Unique sequences
Redundancy check
Filtering by normalized counts
Retained sRNAs
Mapping to tentative contigs derived from male strobili
Mapping to FLcDNAs
miRNA detection
Redundancy check
miRNA candidates
S2 Fig. Flowchart of the sRNA data processing and the miRNA candidates detection.
