# Supplementary figures and images for: Comprehensive analysis of small RNAs expressed in developing male strobili of Cryptomeria japonica
Source: PLoS One. 2018 Mar 12;13(3):e0193665. doi: 10.1371/journal.pone.0193665 (PMC5846777; doi:10.1371/journal.pone.0193665)

## Slide 1
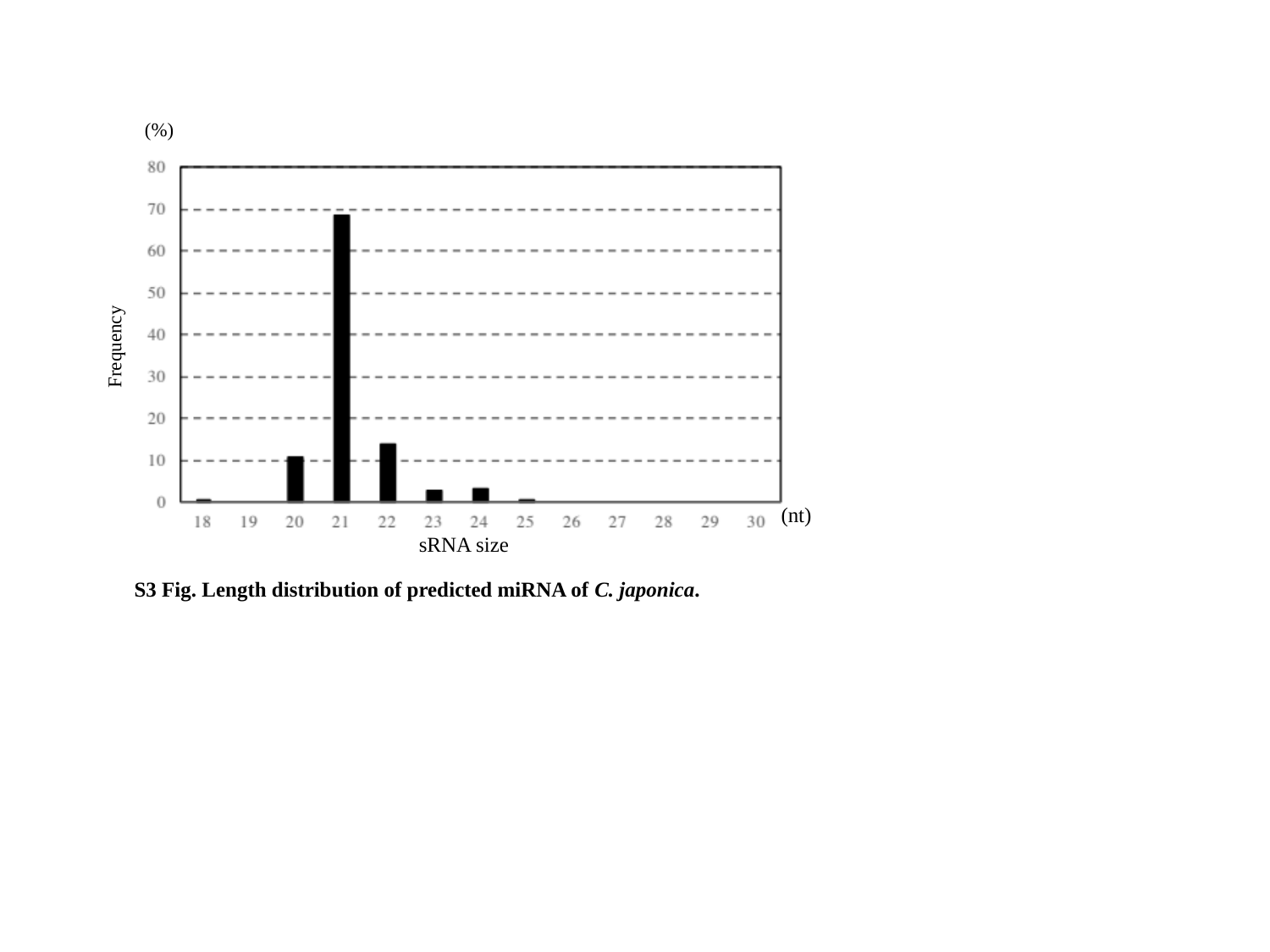

(%)
Frequency
 (nt)
sRNA size
S3 Fig. Length distribution of predicted miRNA of C. japonica.

Supplement: S3 Fig — (PPTX) [file pone.0193665.s003.pptx]
