# Supplementary material for: Comprehensive analysis of small RNAs expressed in developing male strobili of Cryptomeria japonica
Source: PLoS One. 2018 Mar 12;13(3):e0193665. doi: 10.1371/journal.pone.0193665 (PMC5846777; doi:10.1371/journal.pone.0193665)
Supplement: S4 Fig — The dominant mature miRNAs were indicated by green letters. (PPTX) [file pone.0193665.s004.pptx]

## Slide 1
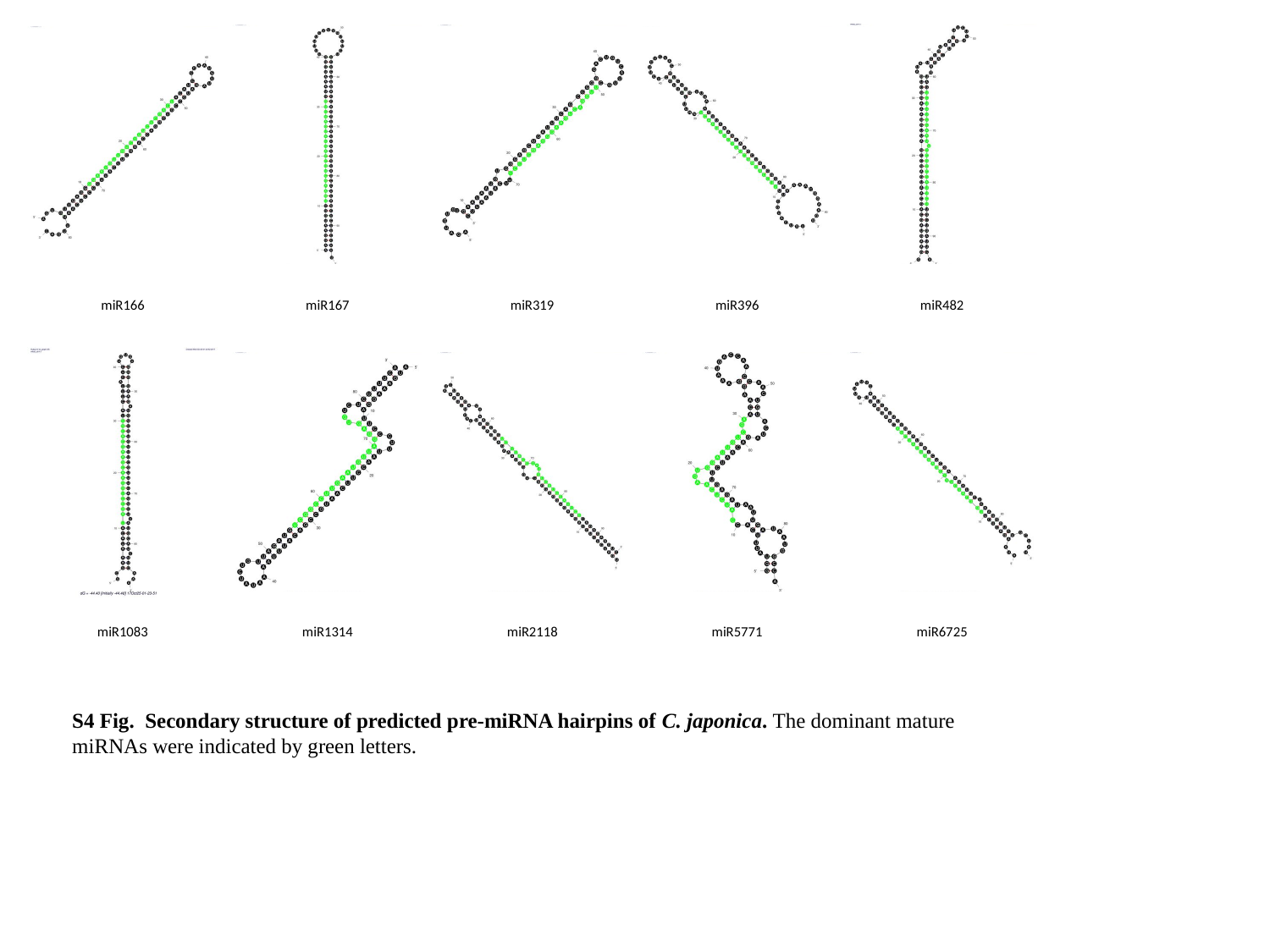

miR166
miR167
miR319
miR396
miR482
miR1083
miR1314
miR2118
miR5771
miR6725
S4 Fig. Secondary structure of predicted pre-miRNA hairpins of C. japonica. The dominant mature miRNAs were indicated by green letters.
