# Supplementary material for: Comprehensive analysis of small RNAs expressed in developing male strobili of Cryptomeria japonica
Source: PLoS One. 2018 Mar 12;13(3):e0193665. doi: 10.1371/journal.pone.0193665 (PMC5846777; doi:10.1371/journal.pone.0193665)
Supplement: S5 Fig — (PPTX) [file pone.0193665.s005.pptx]

## Slide 1
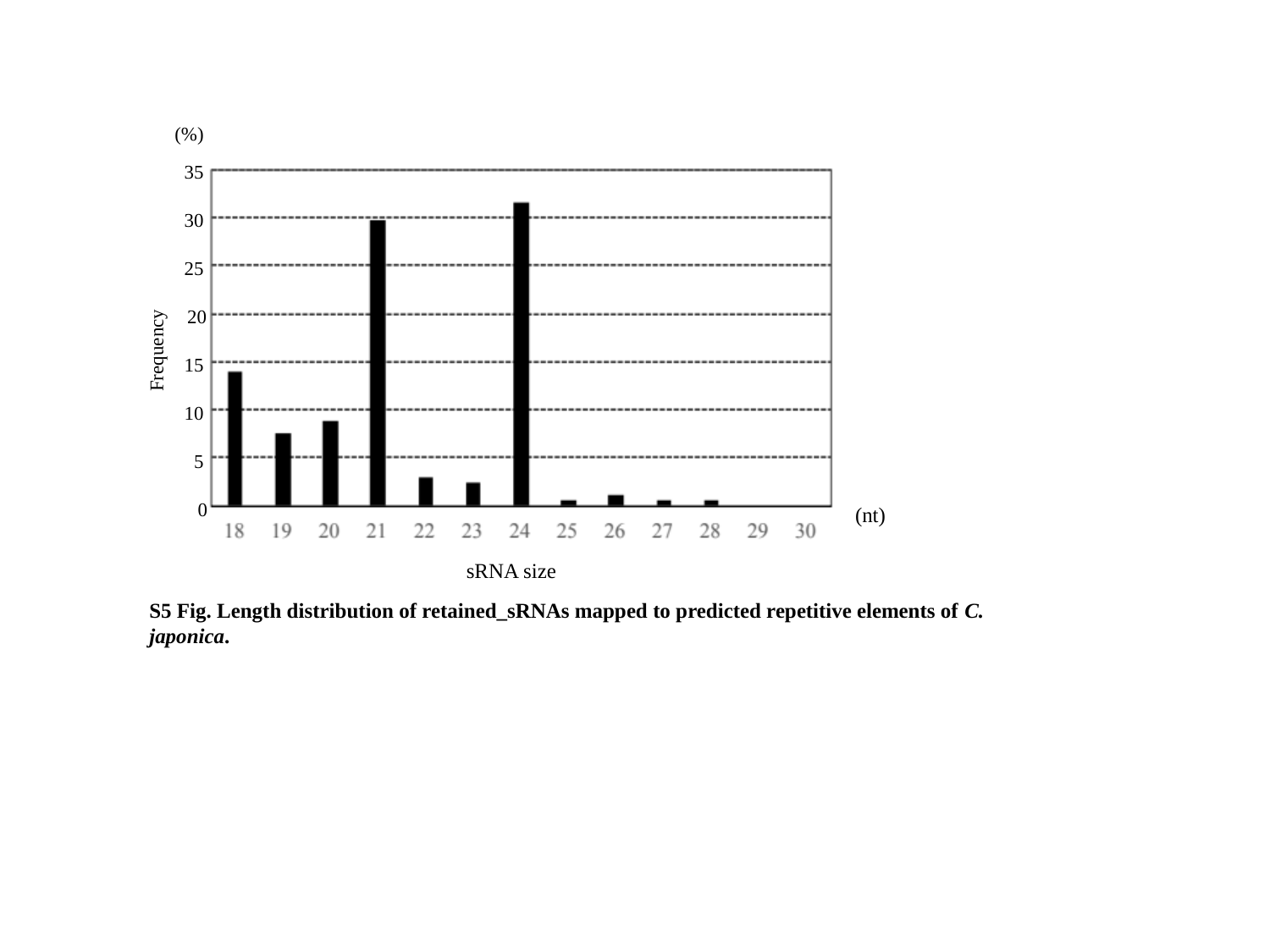

(%)
35
30
25
20
Frequency
15
10
5
0
 (nt)
sRNA size
S5 Fig. Length distribution of retained_sRNAs mapped to predicted repetitive elements of C. japonica.
