# Supplementary material for: Comprehensive analysis of small RNAs expressed in developing male strobili of Cryptomeria japonica
Source: PLoS One. 2018 Mar 12;13(3):e0193665. doi: 10.1371/journal.pone.0193665 (PMC5846777; doi:10.1371/journal.pone.0193665)
Supplement: S6 Fig — Normalized expression level in MS04 sample were arbitrarily set to 1. “qPCR” indicates sRNA expression level based on qRT-PCR and “CPM” indicates the relative expression level based on normalized count data obtained by high-throughput sequencing. For the qRT-PCR result, values are the mean of three technical replicates. Predicted target gene was cjIRX-9 for DEsRNA1 and DEsRNA2, and cjSHT for DEsRNA3. (PPTX) [file pone.0193665.s006.pptx]

## Slide 1
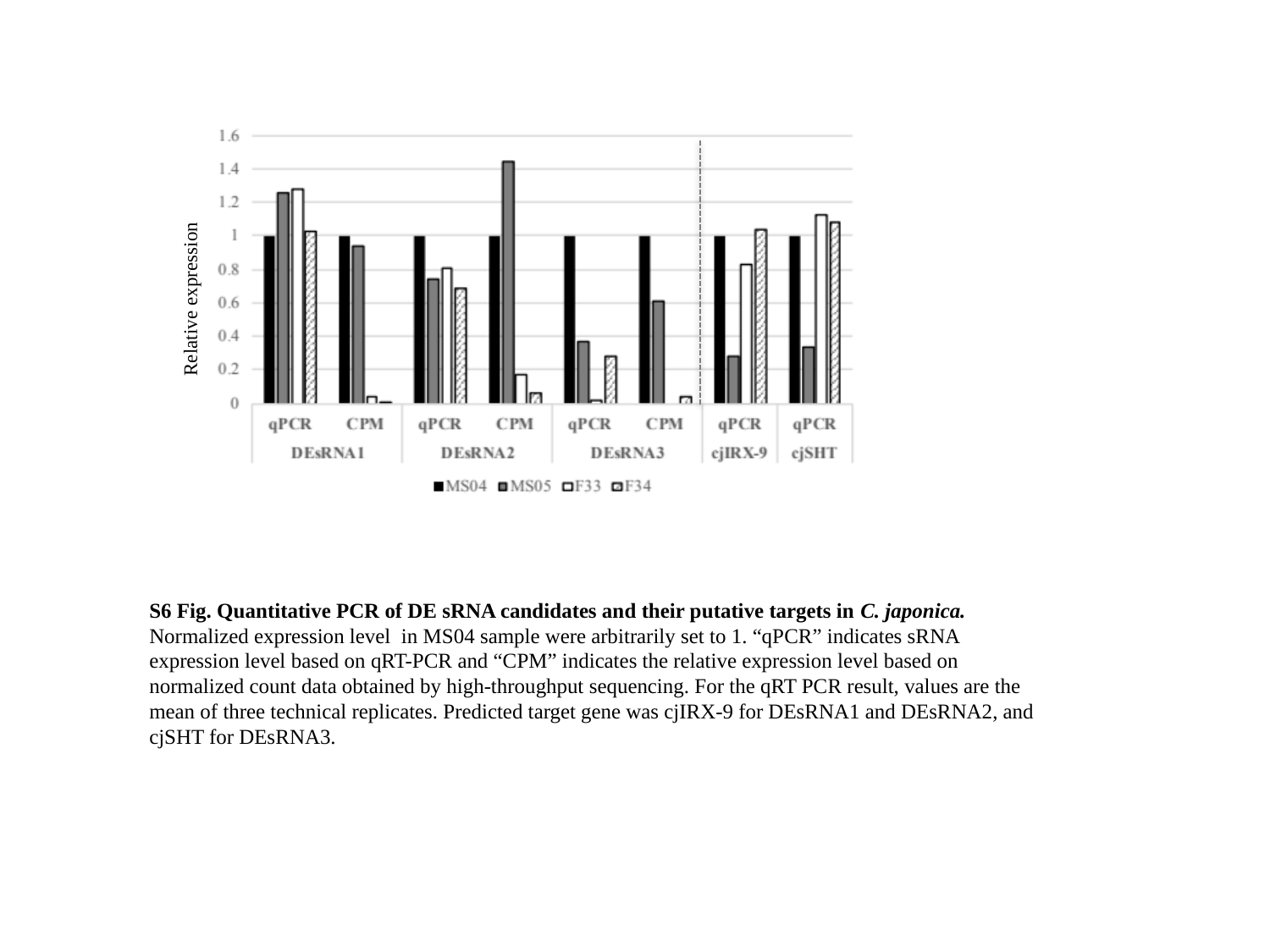

Relative expression
S6 Fig. Quantitative PCR of DE sRNA candidates and their putative targets in C. japonica. Normalized expression level in MS04 sample were arbitrarily set to 1. “qPCR” indicates sRNA expression level based on qRT-PCR and “CPM” indicates the relative expression level based on normalized count data obtained by high-throughput sequencing. For the qRT PCR result, values are the mean of three technical replicates. Predicted target gene was cjIRX-9 for DEsRNA1 and DEsRNA2, and cjSHT for DEsRNA3.
